# The Efficacy of LUCAS in Prehospital Cardiac Arrest Scenarios: A Crossover Mannequin Study

**DOI:** 10.5811/westjem.2017.1.32575

**Published:** 2017-03-14

**Authors:** Robert A. Gyory, Scott E. Buchle, David Rodgers, Jeffrey S. Lubin

**Affiliations:** * Penn State College of Medicine, Penn State Health Milton S. Hershey Medical Center, Hershey, Pennsylvania; † Penn State Health Milton S. Hershey Medical Center, Department of Emergency Medicine, Hershey, Pennsylvania; ‡ Penn State Health Milton S. Hershey Medical Center, Penn State Hershey Clinical Simulation Center, Hershey, Pennsylvania; § Penn State Health Milton S. Hershey Medical Center, Department of Emergency Medicine, Division of Prehospital and Transport Medicine, Pennsylvania; ¶ Life Lion Emergency Medical Services, Hershey, Pennsylvania

## Abstract

**Introduction:**

High-quality cardiopulmonary resuscitation (CPR) is critical for successful cardiac arrest outcomes. Mechanical devices may improve CPR quality. We simulated a prehospital cardiac arrest, including patient transport, and compared the performance of the LUCAS™ device, a mechanical chest compression-decompression system, to manual CPR. We hypothesized that because of the movement involved in transporting the patient, LUCAS would provide chest compressions more consistent with high-quality CPR guidelines.

**Methods:**

We performed a crossover-controlled study in which a recording mannequin was placed on the second floor of a building. An emergency medical services (EMS) crew responded, defibrillated, and provided either manual or LUCAS CPR. The team transported the mannequin through hallways and down stairs to an ambulance and drove to the hospital with CPR in progress. Critical events were manually timed while the mannequin recorded data on compressions.

**Results:**

Twenty-three EMS providers participated. Median time to defibrillation was not different for LUCAS compared to manual CPR (p=0.97). LUCAS had a lower median number of compressions per minute (112/min vs. 125/min; IQR = 102–128 and 102–126 respectively; p<0.002), which was more consistent with current American Heart Association CPR guidelines, and percent adequate compression rate (71% vs. 40%; IQR = 21–93 and 12–88 respectively; p<0.002). In addition, LUCAS had a higher percent adequate depth (52% vs. 36%; IQR = 25–64 and 29–39 respectively; p<0.007) and lower percent total hands-off time (15% vs. 20%; IQR = 10–22 and 15–27 respectively; p<0.005). LUCAS performed no differently than manual CPR in median compression release depth, percent fully released compressions, median time hands off, or percent correct hand position.

**Conclusion:**

In our simulation, LUCAS had a higher rate of adequate compressions and decreased total hands-off time as compared to manual CPR. Chest compression quality may be better when using a mechanical device during patient movement in prehospital cardiac arrest patient.

## INTRODUCTION

The survival rate for patients suffering prehospital cardiac arrest is extremely low, typically in the range of 5–8%.^1^ While there are many reasons for low success rates in prehospital cardiac arrest, two of the most studied and most integral are the performance of proper chest compressions and amount of total hands-off time during cardiopulmonary resuscitation (CPR).

To perform high-quality chest compressions with a minimal amount of hands-off time, they must be done with an adequate depth and rate.^2^ When a provider performs chest compressions on a patient in cardiac arrest, it takes multiple compressions to build up and maintain an adequate intravascular pressure to allow for proper perfusion of the tissues. Additionally, whenever there is a halt in compression application, that pressure is quickly lost and it once again takes time to build that pressure back, leading to large amounts of time with suboptimal perfusion of the heart and brain. Thus, it is critical that interruptions between times that compressions are being performed be kept to a minimum to help increase good patient outcomes. A variety of factors make it difficult to achieve uniformly perfect compressions with minimal hands-off time in the prehospital setting, including needing to move the patient around obstacles, maintaining balance in a moving vehicle, and attempting to perform other Advanced Cardiovascular Life Support (ACLS) measures.^2^,^3^ These tasks can make adequate access to the patient’s chest difficult, which greatly reduces the total number of proper chest compressions and causes large gaps where no compressions are being performed. Moreover, performance of chest compressions is exhausting and most providers will quickly begin to tire, resulting in a decrease in compression quality.^1^

**Figure 1:**
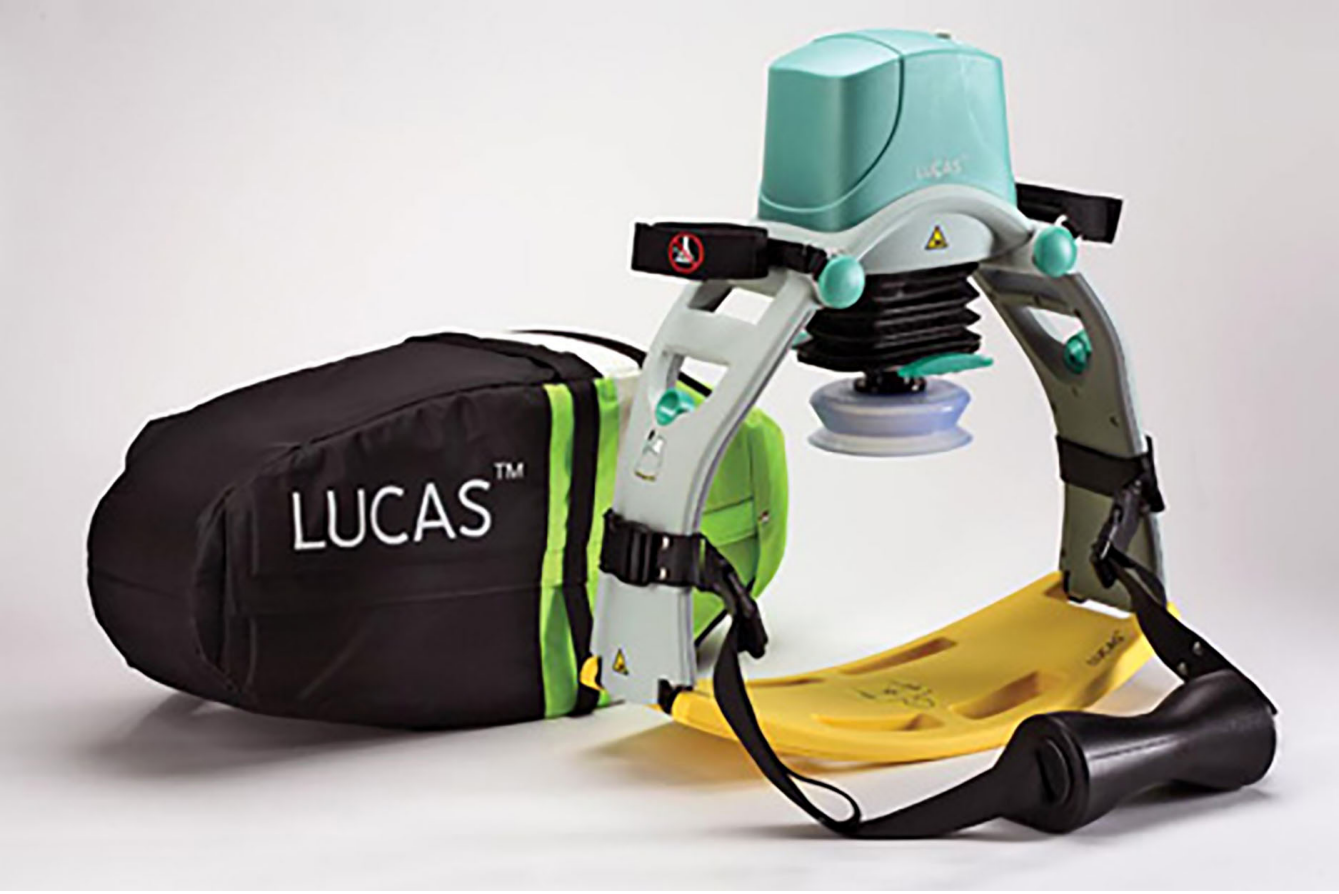
The LUCAS™ chest compression system, a device for mechanical chest compression-decompression. *LUCAS,* Lund University Cardiac Arrest System*.*

Mechanical devices have been designed to perform automated CPR chest compressions on patients so that compressions are not being directly performed by health professionals.^4^ One such device, the Lund University Cardiac Arrest System (LUCAS™), has been in use since the early 2000s. A photo of the device is shown in Figure 1.The debate as to whether the use of mechanical CPR devices results in better patient outcomes, as compared to manual CPR, is currently a heavily debated topic. A number of studies show good rates of return of spontaneous circulation (ROSC) in the field with increased desirable patient outcomes compared to manual CPR.^5^–^10^ At the same time, there are a number of other studies that show no difference between mechanical devices and the efficacy of manual CPR.^11^–^18^

We hypothesized that the use of the LUCAS device in a realistic prehospital cardiac arrest scenario involving transport of the patient would lead to increased quality of CPR, i.e., more in line with American Heart Association (AHA) guidelines, as measured by consistent chest compression rate, a greater compression depth, an increased compression fraction, and full chest recoil as compared to CPR done manually (“manual CPR”). We also hypothesized that deployment of the LUCAS device would not significantly delay time until the first defibrillation of the patient.

**Population Health Research Capsule**

What do we already know about this issue?

Some studies suggest that LUCAS, a mechanical chest compression-decompression device, provides improved CPR in the lab and inpatient settings, but few studies have prospectively assessed prehospital LUCAS use.

What was the research question?

Does LUCAS use in typical prehospital conditions improve CPR and shorten time to critical resuscitation and transport events?

What was the major finding of the study?

LUCAS deployment resulted in improved compression rate and reduced hands-off time, while not delaying defibrillation.

How does this improve population health?

If CPR characteristics and resuscitation events in prehospital arrests are improved with LUCAS, patients may have lower morbidity and mortality following out-of-hospital cardiac arrest.

## METHODS

The study was reviewed and approved by the Pennsylvania State University Institutional Review Board. We recruited subjects from a single hospital-operated advanced life support EMS program working under state-delineated treatment protocols. Teams were composed of one paramedic and one emergency medical technician (EMT). Primary outcomes for the study included time to first defibrillation and better CPR characteristics. For the purposes of power calculation, we based our definition of better CPR on chest compression rate. We expected that a delay of 30 seconds for defibrillation or a compression rate differing by more than 20/min would be clinically significant. Based on these factors, we calculated that enrolling at least 20 participants (10 teams) in the study would provide 80% power to detect a statistically significant difference at α = 0.05.

Upon consenting to participate in the study, subjects were asked to complete a brief questionnaire to obtain demographic information regarding certification level, experience with CPR, and experience with LUCAS. They then completed a 15-minute orientation and training session with the LUCAS device, in which its proper use was demonstrated and the subjects were then permitted to practice and ask any questions about using it.

The study was a crossover controlled mannequin study in which a resuscitation simulation mannequin (Laerdal *Resusci Anne* Simulator Model 150–00001) was fitted with CPR biophysical sensors and attached wirelessly to analytical software designed for the mannequin. This simulation mannequin weighed 36 kg; additionally, because its weight was certainly lighter than that of most real patients, a 14 kg weighted belt was placed around the mannequin to increase its weight during trials to a total of 50 kg. The mannequin was then programmed to present in ventricular fibrillation and was placed on the second floor of a building approximately five miles from the medical center. An EMS crew was asked to respond from the parking lot of the building up to the mannequin. The crew was instructed to go through the state-delineated protocol for cardiac arrest response, including defibrillation pad placement, rhythm identification, one defibrillation, one attempt at airway placement, and performance of manual CPR for at least two cycles prior to any other activity.^19^,^20^

The crew packaged the mannequin in a Reeves litter and carried the mannequin and all equipment down to the waiting ambulance. The path to the ambulance included two stairways, totaling approximately 15 steps, and three narrow hallways. Upon reaching the ambulance, the crew loaded the mannequin onto the ambulance litter and initiated transport to the medical center. The driver of the ambulance was standardized across all scenarios. The crew continued resuscitation efforts until the ambulance pulled into the parking lot of the medical center emergency department (ED). After completion of the scenario, the crew received a 30-minute rest period to recover from the first scenario. They were then instructed to repeat the same scenario, but with use of the LUCAS device in place of manual CPR. The order in which crews completed the two scenarios was randomized.

Mannequin software automatically recorded data on CPR compression rate, compression depth, compression release depth, correct hand position, and time hands off. Specifically, the software provided these data for each CPR characteristic for each compression in a given trial and then automatically calculated descriptive statistics for each trial from the data set. In addition, time elapsed to critical clinical and transport events were marked manually throughout the scenario by an investigator who monitored the conduct of each trial.

### Statistical Analysis

We analyzed the demographic data collected from participants for descriptive statistics only. The descriptive statistics obtained from the mannequin software program were analyzed and inferential statistics were obtained via STATA 9 statistical software (Statacorp, College Station, TX).

Because study data were nonparametric, we used median and percentile comparisons in the data analysis. LUCAS and manual CPR results were compared via the Wilcoxon signed-rank test.

## RESULTS

Thirteen paramedics and 13 EMTs participated in the study. Table 1 summarizes the participant demographics. Table 2 shows the median times to completion of critical transport events for scenarios in which manual CPR and LUCAS CPR were administered. There was no statistically significant difference between the chest compression modalities for time to patient contact, time to CPR initiation, time to placement of the defibrillator pads, time to rhythm identification, or time to arrival at the ED. However, we found that LUCAS took a significantly longer time for arrival of the packaged patient at the litter, time of arrival at the ambulance, and time that transport to the hospital commenced. Median time to first defibrillation was not different for LUCAS compared to manual CPR (132 s vs. 123 s, p = 0.97).

LUCAS was found to perform no differently than manual CPR when analyzing median compression depth, median compression release depth, percent of compressions that were fully released, median time that was hands off in the scenario, and percent of compressions with a correct hand position on the chest. Analyses of these data can be found in Table 3.

**Figure 2A–D:**
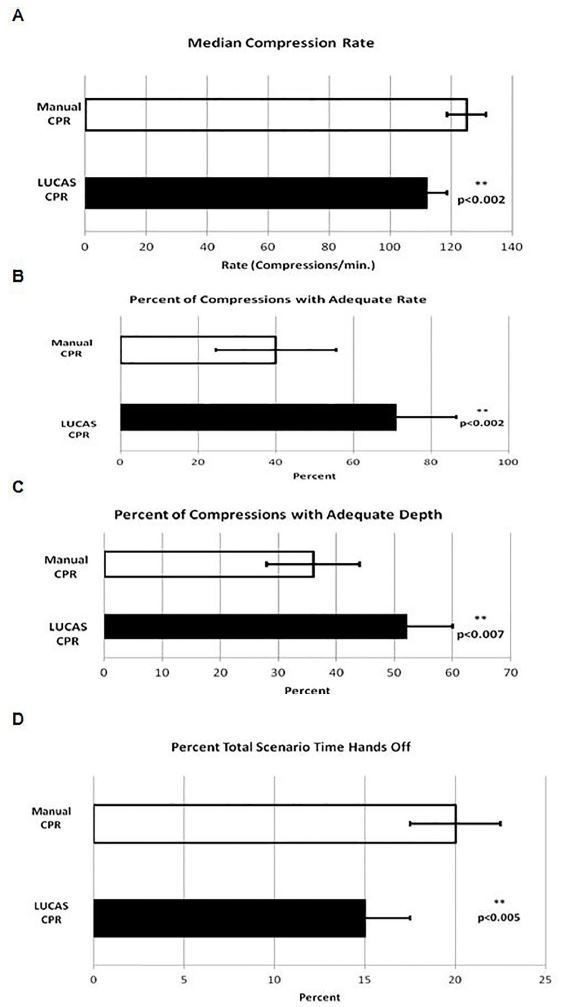
CPR characteristics in which LUCAS performed more optimally than manual CPR. *CPR,* cardiopulmonary resuscitation; *LUCAS,* Lund University Cardiac Arrest System*.*

It was found that median compression rate in the LUCAS scenario (112 compressions/min.) was significantly less than that in the manual CPR scenario (125 compressions/min IQR = 102–128 and 102–126 respectively; p<0.002). The percentage of compression that achieved an adequate rate in the LUCAS scenario (71%) was significantly greater than that achieved in the manual CPR scenario (40%, IQR = 12–93 and 21–88 respectively; p<0.002). Furthermore, the percentage of LUCAS compressions that achieved an adequate depth (52%) was significantly greater than that in the manual CPR scenario (36%; IQR = 29–74 and 25–64 respectively; p<0.007). Finally, the percent total time in the LUCAS scenario that was hands-off time (15%) was significantly decreased with that found in the manual CPR scenario (20%; IQR = 10–22 and 15–27 respectively; p<0.005). Figure 2 shows a graphical analysis of these data.

## DISCUSSION

The AHA has placed a heavy emphasis on improving the quality of chest compressions during CPR; its stance is that while survival from cardiac arrest depends on early recognition of the event and immediate activation of the emergency response system, “equally critical is the quality of CPR delivered.” Proper compressions have been found to lead to increased rates of ROSC both in the prehospital and hospital settings, as well as improved cerebral blood flow and better neurological outcome.^21^–^25^ The increased quality of CPR via the LUCAS device has been already demonstrated in the laboratory and hospital settings. Studies have found that LUCAS provides a compression rate that is consistently able to meet or exceed AHA guidelines. The machine does not become fatigued like a human healthcare provider and so does not reduce its quality of compressions over time.^4^,^19^ In addition, LUCAS allows for less hands-off time during compressions and allows for healthcare personnel to have free hands to perform other tasks such as airway management, IV access, and medications administration. Indeed, when the EMS crew is required to move the patient around tight corners, through narrow hallways, or down multiple stairwells, the device allows for continued compressions during situations in which a patient would almost certainly be receiving no compressions. As the device has continuous access to the patient, lack of provider access to the chest when in awkward locales in the field does not present a barrier to continued CPR. During performance of this study, 100% of crews during manual CPR either completely stopped compressions while moving the patient to the ambulance or else had to not perform compressions while moving the patient and halt transport multiple times to perform a round of compressions. These events were nonexistent when the LUCAS was deployed, with hands-off time occurring during LUCAS CPR only during application of the device and readjustment of the device during slippage. Thus, the patient received continued compression during transport, and the time of transport was not extended due to the crew having to stop movement for a round of compressions. Overall, this process is critical as it allows for a continued maintenance of adequate perfusion pressure to the patient’s brain, heart, and other tissues and does not lead to a loss of that pressure. Most importantly for the EMS system, LUCAS has been shown to be a safe device to employ when on a moving vehicle during emergency transport and has also been shown to be more efficient and effective than manual CPR both in the field and during emergency medical transport, leading to better patient outcomes.^26^–^34^

We were not able to identify any simulation studies that have examined the efficacy of the LUCAS device in the prehospital settings when patients are being moved. The closest study to evaluating prehospital use of LUCAS in a more standard scenario was performed by Blomberg et al., who found that LUCAS did increase the quality of CPR so that compression rate, depth, and other CPR characteristics were in line with current guidelines.^35^ However, this study did not involve movement of the patient, transport via ambulance or assessment of hands-off time, all of which are integral components of real cardiac arrest scenarios.

Importantly, in our study median time to first defibrillation was not significantly different between the two methods of chest compression. These data suggest that the LUCAS device does not delay defibrillation shocks compared to manual CPR; this is consistent with previous literature.^36^–^38^ Compression depth, release depth, and hand position were also not different between the two methods of chest compression. However, when compared to manual CPR, LUCAS provided a compression rate more in line with AHA guidelines and had decreased total hands-off time. Interestingly, the compression rate during manual CPR was found to have a median that exceeded the recommended compression rate by the AHA. This result was surprising because we expected subjects to be more fatigued during manual CPR, resulting in a lower median rate than with LUCAS. The cause of this result is unclear, although it might be related to the Hawthorne effect or problems in original CPR training. More research into this area may be warranted in the future.

There were no significant differences among many of the marked critical times in transport of the patient to the ED. This suggests that LUCAS neither delays nor reduces a large fraction of the transport time compared to manual CPR. When considering time to arrival at the patient, CPR initiation, defibrillator placement, and rhythm recognition, these data intuitively make sense as they are not related directly to whether or when the LUCAS may be deployed in a scenario. Regarding final arrival at the ED, one would expect that the overall scenario would take a shorter time with the LUCAS device as the crew would not have to continually halt transport for compression rounds; yet, no significant difference was found between the scenarios.

This finding is most likely related to three separate phenomena. First, the crews who were unfamiliar with the LUCAS device appeared to struggle with proper deployment and assembly around the patient; this delay, while not measured, may have offset any of the time saved during the rest of the transport. Another consideration is that some of the crews, during the manual CPR trials, chose not to halt transport to the litter multiple times to engage in CPR rounds as would normally be recommended. It was not measured how many crews transported in this manner, nor how long a delay was incurred for crews that did halt for CPR, but continuous transport down to the litter, though increasing scenario hands-off time, decreased total transport times in a manner that rivaled the time saved with LUCAS. Finally, all crews followed the Pennsylvania state EMS protocols, which required at least two full rounds of manual CPR prior to LUCAS deployment. While realistic and true to required standard of care for these crews, it certainly delayed transport time in the LUCAS trials that would likely not have been seen if LUCAS had been permitted to be deployed immediately. Indeed, many states would have allowed for immediate LUCAS deployment.

Further investigation into transport times without the prerequisite rounds of manual compressions is warranted. It is important to note that time to arrival at the litter and time to initiation of transport to the ED were found to be significantly increased when LUCAS was used. It may be that the aforementioned alleviation of needing to halt transport for a round of compressions makes it easier to transport the patient, but may not have been offset by the other variables as stated above. To better determine the full effects on critical transport events, further study will be required that can control for these variables. Whether either mode of chest compression delivery had a transport time difference that was of clinical significance is unclear.

The use of the LUCAS may contribute to improved safety. The most obvious example of this is that crew members were able to sit safely seat belted during transport when LUCAS compressions were being done, but had to stand in the moving ambulance while doing manual CPR. In addition, crews had time to perform other tasks during transport with LUCAS because their hands were not occupied with manual CPR. This extra time may allow providers the chance to complete such things as placing an advanced airway, starting additional intravenous lines, giving more timely medications, and contacting the receiving hospital to give a report. The crew members were able to perform these tasks in a calmer and less hurried manner with LUCAS compared to manual CPR, which might suggest that LUCAS allows for more time for crews to think clearly and perform optimally during patient care.

## LIMITATIONS

There were several limitations to this study. First, due to time, resource, and financial constraints this study included a single EMT/paramedic team in the cardiac arrest response. While the use of LUCAS allowed for better CPR characteristics and increased opportunity for ACLS milestones, most real-world cardiac arrest responses will include either fire support or at least one other EMS team. Thus, it cannot be determined from this study that the increased ability of crews to complete ACLS milestones would be due solely to LUCAS in a real arrest response, as there would be more personnel available to perform compressions and would allow the EMS team to focus on activities not related to compression performance.

Most participants in the study had not received any training or practice on LUCAS prior to that given in the study. This inexperience may have led to uncertainty and hesitancy when using the LUCAS under pressure in the study scenario, which may have led to falsely increased overall scenario times due to the hesitancy and not to deployment of LUCAS itself. The skin of the mannequin did not consistently allow for realistic contact of the plunger of the LUCAS device, which led to some slippage of the device off of the midsternal region in a few scenarios.

In addition, during the LUCAS scenarios crews were highly variable in the amount of time before switching from the original rounds of manual CPR to compressions delivered by LUCAS (range: 136–378s); because this was not standardized among all crews, it added some additional variability to performance and may once again have increased overall scenario time in a way that had nothing to do with LUCAS. Additionally, the 30-minute rest period may not have been of sufficient length to allow for fatigue to be addressed between scenarios for crew members; there seemed to be some residual fatigue across several crews during the second scenario which would again have increased overall scenario time.

Another potential factor that may have increased overall scenario time not directly related to the LUCAS device was the use of the state of Pennsylvania EMS protocols for cardiac arrest response. To allow for realism in this study, the state protocols were adhered to as they should be in a real response; however, the state requires at least two rounds of manual CPR before LUCAS or another mechanical device may be deployed. This requirement may have falsely increased overall scenario time as it prescribed close to two full minutes in which the crew was not permitted to deploy LUCAS or focus on other activities other than compression performance. In other states, these requirements do not exist and LUCAS may be deployed immediately. Thus, the use of the Pennsylvania state protocols, while realistic, may have added extra time to the scenario that was not secondary to the actual LUCAS device.

Due to time, cost and safety, some realistic aspects of the scenario had to be sacrificed. These aspects included lack of lights and siren transport of the crew to the hospital and an allowed pre-study walkthrough of the path from the ambulance to the mannequin for the crews. Moreover, the total weight of the mannequin and the weighted belt was 50 kg. While this additional weight was used to attempt to add a bit more realism to the study scenario, most patients that EMS will come in contact with are significantly heavier than this. Thus, the overall patient package may still have been significantly lighter than a real patient and may have made traversing the overall scenario less difficult than would be seen in a real response. Finally, due to the fact that this was a mannequin study, it is unclear what the real effects would be on patient outcomes. As such, while this study can speak to the physical parameters of completing an arrest scenario, it can only be used as a bridging study that will lead from isolated CPR-performance assessment without realistic arrest scenarios to studies assessing real deployment in patient care. Further research that includes deployment in real cardiac arrest scenarios will be imperative to determine patient outcomes.

## CONCLUSION

As previously stated, there are data showing that LUCAS is very effective in prehospital cardiac arrest and patient outcomes and that the device is safe for patient use and does not lead to undue patient injury.^39^–^41^ However, not enough data on real patients exist; thus, this area is clearly ripe for future work.

In this mannequin study attempting to assess the efficacy of the LUCAS device in a realistic prehospital cardiac arrest scenario, LUCAS provided chest compressions that were more consistent with AHA standards without creating delays to critical resuscitation tasks such as defibrillation. Moreover, total hands-off time was reduced in LUCAS scenarios, which would lead to maintenance of adequate perfusion pressures and may afford better overall patient outcomes. The effect of patient movement on chest compression quality must be considered as the use of mechanical CPR devices is deliberated by EMS agencies.

## Figures and Tables

**Table 1:** Demographic data of emergency medical services participants in a study of the use of a mechanical chest compression-decompression device vs. manual CPR.

Demographic	EMT-B (n=13)	EMT-P (n=13)
Mean number of years in EMS (range)	13.5 (6–27)	19.4 (4–47)
Number who received LUCAS training in CPR course	1	2
Current CPR instructor	2	4
Mean previous LUCAS training sessions (range)	2 (1–8)	2 (1–12)
Mean estimate of times performed manual CPR (range)	50 (10–100)	95 (15–230)
Mean estimate of times LUCAS used (range)	2 (0–15)	2 (0–10)

*CPR,* cardiopulmonary resuscitation; *EMS,* emergency medical services; *EMT-B,* emergency medical technician-basic; *EMT-P,* emergency medical technician-paramedic; *LUCAS,* Lund University Cardiac Arrest System*.*

**Table 2:** Median time to completion of critical transport events.

Time-stamped event	Median time with manual CPR (s) (IQR)	Median time with LUCAS (s) (IQR)	p-value
Patient contact	31 (29–33)	32 (31–34)	0.27
CPR initiation	66 (45–71)	62 (56–76)	1.0
Placement of defibrillator pads	100 (86–110)	105 (94–111)	0.22
Rhythm identification	106 (103–129)	120 (103–130)	0.97
Defibrillation performed	123 (108–135)	132 (113–141)	0.97
Arrival at litter	369 (338–412)	422 (312–493)	0.006*
Arrival at ambulance	538 (493–559)	622 (425–753)	0.06*
Begin transport	565 (517–610)	664 (454–805)	0.03*
Arrival at ED	1436 (1369–1468)	1411 (1353–1478)	0.21

*CPR,* cardiopulmonary resuscitation; *LUCAS,* Lund University Cardiac Arrest System*; ED,* emergency department.

**Table 3:** Analysis of chest compression characteristics between the LUCAS mechanical device and manual CPR.

Chest compression characteristic	LUCAS CPR (IQR)	Manual CPR (IQR)	p
Median compression depth (mm)	36 (35–38)	37 (35–48)	0.83
Compressions fully released (%)	93 (77–96)	78 (72–88)	0.67
Median duration of hands off event (s)	7 (5–9)	9 (7–12)	0.86
Compressions with correct hand position (%)	91 (78–100)	96 (88–99)	0.83

*CPR,* cardiopulmonary resuscitation; *LUCAS,* Lund University Cardiac Arrest System*.*
